# Assessment of potassium current in Royan B_1_ stem cell derived cardiomyocytes by patch-clamp technique

**Published:** 2009

**Authors:** H. Sadraei, S.R. Abtahi, M. Nematollahi, K. Karbalaie, F. Karamali, H. Baharvand, M.H. Nasr-Esfahani

**Affiliations:** 1 *Department of Pharmacology & Toxicology and Isfahan Pharmaceutical Sciences Research Center, School of Pharmacy and Pharmaceutical Sciences, Isfahan University of Medical Sciences, Isfahan, I.R.Iran*; 2 Department of Cell and Molecular Biology, Royan Institute for Animal Biotechnology, ACECR, Isfahan, I.R.Iran

**Keywords:** Royan B_1_ stem cells, Cardiomyocytes, K^+^ current, Tetraethylammonium, Membrane potential, Patch-clamp

## Abstract

Embryonic stem cells are capable of differentiating to variety of cell tissues including cardiomyocytes. This developmental change is accompanied with a great deal of ion channel expression and functions. Mouse stem cell derived cardiomyocytes were prepared and separated to yield isolated single cell suspension for cell current recording. In the present study some properties of the K^+^-current in Royan B_1_ stem cell derived cardiomyocytes were investigated using whole cell patch-clamp technique. When the holding potential was - 60 mV, in some cells a major outward current was elicited by square depolarizing pulses from -60 mV to +50 mV. This outward current was sustained for the duration of 300 ms test pulse. The sustained outward K^+^ current was inhibited by tetraethylammonium (10 mM) indicating the activity of Ca^2+^ activated K^+^ channel in these cells. In some of the cells with 0.2 mM 3,ethylene glycol-bis (β-aminoethyl ether) N,N,N^`^,N^`^-tetraacetic acid in the pipette, only a very small outward current was recorded which suggests that in these cells the voltage activated K^+^ channels is either absent or if existed it is not fully functional. Other cells were in far between, indicating that voltage activated K^+^ channels are developing in these cells but it is not yet fully functional. In conclusion, we have identified functional large conductance Ca^2+^ activated K^+^ channel in Royan B_1_ stem cell derived cardiomyocytes.

## INTRODUCTION

Mouse embryonic stem cells (ESCs) are pluripotent cell lines, initially derived from the inner cell mass of mouse blastocyst-stage embryos (1–3). Cells of the inner cell mass normally give rise to the three germ layers from which all tissues and organs of the body develop. However, these cells can also be grown in culture, as embryonic stem cells, and can be used to study the processes of development and differentiation (1). One of the main characteristics of ES cells is their ability to proliferate endlessly and to maintain a pluripotent state. This requires special culturing methods and reagents. Removal of the undifferentiated cells from their feeder layer and their culture in suspension leads to the formation of embryo-like aggregates, known as embryoid bodies, which may spontaneously differentiate into cells with cardiomyocytic characteristics in the outgrowths of embryoid bodies (4,5). Studies have demonstrated that ESCs derived cardiomyocytes express multiple cardiomyocyte-associated structural proteins and transcription factors; respond to a number of pharmacological reagents; display types of action potentials that are similar to embryonic nodal, atrial, and ventricular cardiomyocytes; show proliferation capacity and express proteins that are typical for early-stage cardiomyocytes (2).

Undifferentiated stem cells exhibit no electrical activity, are unable to generate action potentials, and reveal only slight linear current voltage relations (6). The various shapes of action potentials in ES cell-derived cardiomyocytes of different developmental stages are well correlated with the expression of specialized types of ion channels (5). In cardiomyocytes of an early differentiation stage, the primitive pacemaker action potentials are generated by two main types of ion channels, i.e. voltage activated L-type Ca^2+^ channels (I_Ca_) and transient outward K^+^ channels (I_K_,_to_). Terminal differentiated cardiomyocytes express various additional types, including voltage-dependent Na^+^ channels (I_Na_), delayed outward rectifying K^+^ channels (I_K_), inward rectifying K^+^ channels (I_KI_), muscarinic acetylcholine-activated K^+^ channels(I_K_,_Ach_) and hyperpolarization activated pacemaker channels (I_f_) (7,8). Most electrophysiological and pharmacological properties of the ionic currents of ES cell-derived cardiomyocytes are similar to those previously described for adult (9).

Contractility is a primary cardiomyocytes function (10). Excitation-contraction coupling is the mechanism that connects the electrical excitation with cardiomyocyte contraction (10). One of the obvious changes in the membrane potential of cardiomyocytes is hyperpolarization of resting membrane potential. Changes in the resting membrane potential result from opening or closing of ion channels (10–13). Significant changes in voltage gated ion channels have been recognized in developing heart. In rat ventricular cells, ion channel development is characterized by the prenatal enhancement of L-type Ca^2+^ channel expression and postnatal increase in transient outward K^+^ currents (14,15). During cardiac development, there is a reciprocal relation between cardiac and morphogenesis and contractility. In the early embryonic myocardium, the sarcoplasmic reticulum is poorly developed, and Ca^2+^ channels are critical for maintaining both contractility and excitability (10,15).

Embryonic cardiomyocytes are not only capable of generating action potential and contraction but they also induce spontaneous pacemaker activity (10). Ion channels are extensively expressed in these cells and they have important roles in maintaining physiological functions. Inward L-type and T-type Ca^2+^ channels, and outward large-conductance Ca^2+^ activated K^+^ channels, delayed rectifier and transient K^+^ currents are the ion channels that have been described in cardiomyocytes (15,16).

Embryonic mouse stem cells are capable of differentiating to variety of cell tissues including neurons and cardiomyocytes (17,18). These developmental changes are accompanied with a great deal of ion channel functions; because during this process a nonexcitable cell is converted into an electrically excitable cell that can fire action potential (15). Large-conductance Ca^2+^ activated K^+^ channels are present in electrically excitable and nonexcitable cells and have important physiological role in the regulation of membrane potential. These channels have been identified in mouse cardiomyocytes and have a significant role in regulation of cellular excitability (15,19).

Embryonic stem cell derived cardiomyocytes express cardiac-specific genes and ionic currents (20). Studies of ionic channel expression during early period of development of embryos are limited because of the small size of the embryonic heart and the lack of permanent cells lines to model the earliest stage of cardiomyogenesis. However, study of the early stages of cardiomyogenesis is possible by using cardiomycocytes differentiated *in vitro* form pluripotent mouse embryrinoic stem cells (20). Unlike permanent cardiac cell line from embryonic rat embryo, stem cell derived cardiomyocytes express all types of cardiac ionic channels. Patch-clamp study is an electrophysiological technique that can detect the expression of specific channel during cell development in live cells (20–22). Large conductance Ca^2+^-activated K^+^ current is less documented in the stem cell derived cardiomyocytes. The present study in addition to describing development of cardiomyocytes from stem cells, characterizes the development of large conductance Ca^2+^ activated K^+^ currents in Royan B_1_ cardiomyocytes, derived from mouse embryonic stem cells, using whole cell patch-clamp technique.

## MATERIALS AND METHODS

### Mouse embryonic stem cell culture

Royan B_1_ stem cell derived cardiomyocytes were prepared as described by Baharvand *et al* (2,23).

### Preparing fibroblast feeder layer

Frozen mouse embryonic fibroblast (Royan NMR1) were taken out of liquid nitrogen, thawed and incubated in semi-complete medium (see solutions) in tissue culture flask (Falcon T150) at 37 °C in 5% CO2 with 85-100% humidity for 4 days. Then, mitomycin C (Sigma M-0503) was added to the medium to a final concentration of 10 µg/ml and incubated for further 1.5 h to keep the cells in an undifferentiated state. The flask was then rinsed twice with 5 ml of phosphate buffer solution (PBS). Thereafter, the cell culture was trypsinized by covering the surface of the cells with Trypsin (0.05%)/EDTA for 3 min. By adding equal volume of medium, the trypsin was neutralized and cell suspension was formed by pippeting. The cell suspension was further diluted and an aliquot of the cell suspension was transferred to tissue culture flask (Falcon T25) with gelatin-coated (0.1%) surface for further incubation. A day later cells were attached to the flask and formed the fibroblast feeder layer (24).

### Stem cell differentiation into cardiomyocyte

To culture stem cells on fibroblast layer, the semi-complete medium was removed and replaced with complete medium [semi-complete medium with added leukemia inhibitory factor (LIF 1000 iu/ml) to prevent differentiation]. A vial of stored mouse embryonic stem cells line (Royan B_1_ derived from C57BL/6 mouse strain) was removed from liquid nitrogen and thawed and poured into the above prepared feeder plate and incubated at 37 °C for two days. Every day the cell cultures were viewed under microscope for normal colony, and cell medium was removed by aspiration and replaced with fresh medium.

Embryonic stem cells were routinely passaged every two days. To do this, the medium was removed and cells were washed twice with 2 ml of PBS (without bivalent cations). About 2 ml of 0.5 mM ethylene glycol-bis (β-aminoethyl ether) N,N,N^`^,N^`^-tetraacetic acid (EGTA) was added to each tissue culture flask until the connection of the colony cells became loose without shaking the flask (about 2 min). The EGTA was removed and 1 ml of trypsin/EDTA solution was added to each flask until the colonies became afloat by flicking the plate (about 3 min). The colonies were dissociated into single cells by pippeting. Then, by adding 1.5 ml of the culture medium, the trypsin was inactivated. An appropriate volume of the cell suspension was transferred into several flasks for further incubation and the medium was changed next day. A day later, the cells were trypsinized as described above, and colonies were dissociated into single cells by pippeting. One ml of semi-complete medium [without leukemia inhibitory factor (LIF)] was added to inactivate the trypsin/EDTA and further pippetted to complete the cell separation. Further 8 ml of semi-complete medium was added to this single cell solution. Cells suspension was then transferred into a 150 ml gelatin-coated cell culture flask and incubated for 1.5 h. During this time the fibroblasts adhered to the dish while the stem cells remained in the suspension. The suspension was then gently transferred into a centrifuge tube and centrifuged at 1800 rpm for 10 min. The supernatant was discarded and the pellet was resuspended in 1 ml of culture medium and the cells were counted using hemocytometer counting chamber.

Stem cell suspension with a definite number of cells (800) in 20 µl drop was deposited on inverted petri dish cover in semi-complete medium containing 0.1 mM vitamin C (Sigma A4544) for two days. Within two days the resultant hanging drops formed the embryoid bodies

After two days of incubation by using yellow tip under stereomicroscope, 50-70 embryoid bodies were transferred into nonadhesive bacteriological grade petri dishes (6 cm wide, Grainer) containing 5 ml of semi-complete medium plus vitamin C, and further incubated for five days. Every other day the cell cultures were inspected. If the medium color had not changed, only vitamin C was added, otherwise the medium was changed. On day seven, the embryoid bodies were removed and plated separately into 1% gelatin coated wells of a 12-well culture plates (TTP, Switzerland) containing semi-complete medium for cell differentiation into cardiomyocytes. After 4-5 days incubation cell beating could be viewed under microscope, which confirms differentiation of stem cells into cardiomyocytes (2,23,25).

### Cell preparation for patch-clamping

Only cell cultures with active beating were used for patch-clamp recording. On the day of experiment, the medium was removed by aspiration and cells were washed twice with PBS. Trypsin/EDTA solution was then added in a volume that just covered the entire cell surface and culture plates were incubated for 3 min at 37 °C in 5% CO_2_ with 98-100% humidity. Thereafter, an equal volume of semi-complete medium was added to neutralize the enzyme. Then, the entire cell culture was mechanically agitated by gentle aspiration and expelling of the cells in and out of a glass pasteur pipette to isolate the cells. The cell suspension was then transferred into a conical tube and centrifuged for 10 min at 1800 rpm. Thereafter, the enzyme and medium mixture was decanted and the pellet was resuspended in fresh PBS. The cell suspension was then recentrifuged twice in PBS to remove any excess enzyme. In the last stage, the cell pellet was resuspended in N-[2-hydroxyethyl] piperazine-N^`^-[2- ethanesulphonic acid] (HEPES) solution for patch-clamp recording (see solutions). Cell suspensions were kept at 4 °C and recordings were made from cells within 10 h. All above processes were performed under laminar flow cabinet to exclude contaminating microorganism.

### Recording

An aliquot of cell suspension (0.2 ml) was placed into the recording chamber which was mounted on the stage of the phase-contrast microscope. The cells were left for a few min to settle on to the bottom of the chamber before perfusion with bath solution was started at about 2 ml/min. Solutions were perfused by gravity flow and removed by aspiration into a waste container connected to a vacuum. All drugs were applied via the bath solution by switching the inflow to another reservoir.

Membrane currents were recorded using the whole-cell configuration of the patch-clamp technique (26). Patch pipettes were pulled from borosilicate hematocrit glass capillaries (1.5 mm O.D. × 0.85 mm I.D.; Hirschmann Laborgerate) using a two-stage pipette puller (L/M-3P-A patch-clamp puller). The tip of each pipette was viewed under a microscope (Zeiss inverted microscope) and then fire-polished (L/M-CPZ-101 Coating and Polishing System). Pipettes were filled with pipette solution and secured into an electrode holder with a silver-silver chloride wire making contact with the pipette solution. A silver-silver chloride ground electrode was placed directly into the bath solution to complete the circuit.

Cells were viewed using an inverted microscope (Leica Microscope DM-IIRB) placed on an air table (T-250 Vibration Isolation Laboratory table). A hydraulic micromanipulator (MHW-3-1) and a course manipulator (Narishige) fixed onto the side of the microscope were used to lower the electrode into the bathing solution and onto the cell membrane. Once the pipette was in contact with the bathing solution the pipette current automatically was zeroed. A 10 mV step potential was then applied to the pipette and current flow through the pipette was measured on the oscilloscope and the pipette resistance calculated using Ohm’s law (V = IR). Pipettes with resistance of 1-4 MΩ were used for recording (26–28). The pipette tip was then placed onto the surface of a cell and a high resistance seal (giga-seal) was formed between the pipette glass and the cell membrane by applying gentle suction. Further suction was then applied to rupture the membrane within the pipette to allow access to the whole-cell. The holding potential was usually maintained at -50 or -60 mV and test voltage-steps were applied for 300 ms every 10 s. In addition, whenever possible, resting membrane potentials were recorded by switching the mode switch on the current monitoring panel of amplifier to current clamp mode.

### Current sampling and analysis

Membrane currents were recorded using a patch-clamp amplifier (L/M-EPC-7) which had a feedback resistor of 500 MΩ. Voltage-pulses were generated by a stimulator in the amplifier via a computer program. Signals filtered by a 10 KHz, 3 pole Bessel filter inserted into the current monitor pathway, viewed on a oscilloscope (Hameg HM 303-4) and capture online to a computer (IBM compatible Pentium 166 Mhz MMX). The software for data capture and analysis was Win Tida data acquisition and analysis software for windows (List-Electronic). A record consisted of a series of digital samples digitised by an analog-to-digital (A-D) convertor (ICT-16, Data Acquisition Interface) at a defined rate.

### Solutions and drugs

#### Cell culture media

Incomplete medium was prepared by adding 2 ml pencillin/streptomycin (1%; GIBCO 15070-063), 2 ml non-essential amino acids (0.1 mM; GIBCO 11140-035), 200 µl β-mercaptoethanol (0.1mM, GIBCO 3135-010), and 30 ml ES-Fetal Bovine Serum (15%; GIBCO 10439-024) into 200 ml of Dulbeco’s modified Eagl’s medium (Knockout^TM^D-MEM; GIBCO 10829).

For preparation of semi-complete medium, 500 µl of L-glutamate (2 mM; GIBCO 25030-024) was added to the incomplete medium (above).

Complete medium was prepared by addition of 50 µl of leukemia inhibitory factor (1000 iu/ml; LIF 10^7^ unit, Millipore ESG1107) to 50 ml of the semi-complete medium.

PBS: 9.5 g of PBS powder without bivalent cations (GIBCO 21600-010) was dissolved in 1000 ml of double distilled water.

Trypsin/EDTA solution: GIBCO (25300-054) containing 0.5 g/l of trypsin and 0.2 g/l of EDTA. 4Na in Hank’s Balanced Salt solution without Ca^2+^ and Mg^2+^..

#### Patch-clamp solution

Dispersion solution (mM); NaCl 126, KCl 6.0, HEPES 10, CaCl_2_ 0.05.

Bath solutions (mM): Ca^2+^ bath solution; NaCl 135, KCl 5.0, CaCl_2_ 1.5, MgCl_2_ 1.2, HEPES 10, glucose 10. Both dispersion and bath solutions were titrated to pH 7.4 with NaOH (1 M).

Pipette solutions: K^+^ current recording (mM); KCl 130, MgCl_2_ 2, HEPES 10, Na_2_ ATP 3, EGTA 0.2. The pipette solution was titrated to pH 7.4 with KOH (1 M), filtered through a 0.2 µm pore syringe filter (Acrodisc, Gelman Sciences) and stored and frozen in 1 ml vials.

Tetraethylammonium chloride (Sigma) was initially dissolved in distilled water as 100 mM stock solutions, and then diluted in bath solution to give 10 mM TEA in the bath solution. Unless stated, all the chemicals were from Merck.

### Data analysis

To analyze the records for each signal, a fixed zero level was defined. The voltage signal was measured relative to zero volt, while the current zero level was calculated from a portion of each record by averaging points on a defined cursor position at the holding potential level prior to the test voltage step. The current generated by stepping from the holding potential was analyzed by measuring the averaged current in a defined portion of the record.

The mean and standard error of mean (S.E.M) of the current for each test potential was calculated and plotted against the voltage (I/V curve). If appropriate the data were statistically compared for differences using analysis of variance (ANOVA) and or Student’s t-test.

## RESULTS

### Stem cell derived cardiomyoctes

**Fig. 1:**
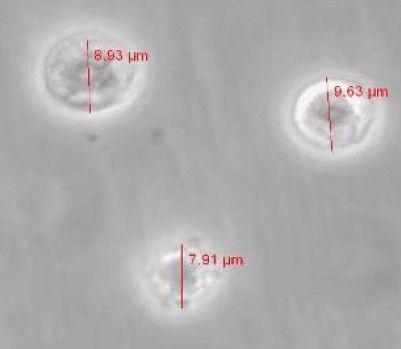
Isolated Royan B_1_ stem cell derived cardiomyocytes on day 8. The cell size ranged from 6 µm to 18 µm.

After 4-6 days of incubation stem cells started beating which confirms that they are differentiated into cardiomyocytes (2,16,23). However, not all the cells have had contractile activity. Enzymatically separated stem cell derived cardiomyocytes had a round shaped appearance with various sizes (9.4 ± 0.44 µm; n=27). The healthy cells looks phased bright (see Fig. 1) and could be used for seal formation.

Most of the cells remained healthy in the fridge but at room temperature they didn’t survive for long time. It was impossible to form a giga-seal on damaged cell. Therefore, stable giga-seal formation itself was confirming recording from a healthy cell. These cell had a resting membrane potential ranging from -30 mV to -66 mV (mean 43 ± 5.4, n=228).

### Current recording

**Fig. 2:**
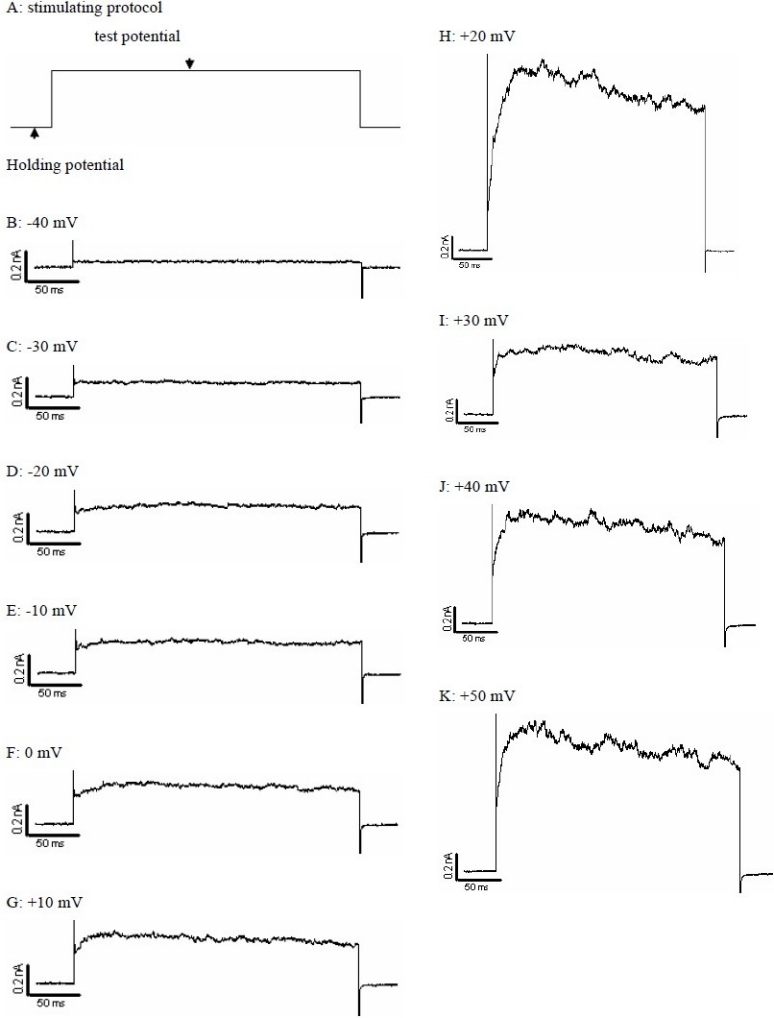
Original sustained outward current recorded with 0.2 mM EGTA in KCl recording pipette and Ca^2+^ bath solution. A: shows the voltage stimulation protocol. The sustained current was elicited by depolarizing from holding potential of -60mV to test potentials of -40, -30, -20, -10, 0, +10, +20, +30, +40 and +50 mV for 300 ms (B-K). The initial spike indicates the cell junction potential. The fluctuating outward current superimposed on the sustained current is characteristic of the current produced by large conductance Ca^2+^ activated potassium channels.

**Fig. 3:**
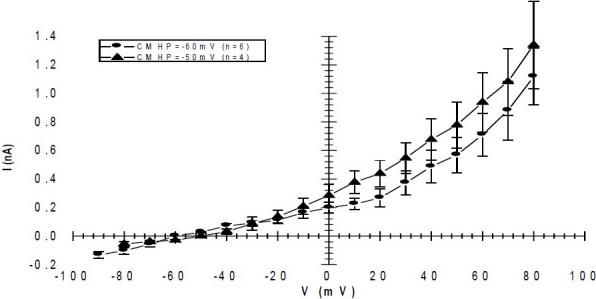
Comparison of current-voltage (I/V) curve for outward sustanied current recorded from stem cell derived cardiomyocytes at holding potentials (HP) of -60 mV and -50 mV. The sustained current was elicited by depolarizing from holding potential to various test potentials for 300 ms. All the values are mean ± S.E.M. Mean current amplituedes were measured relative to holding current. There are no statistically significant differences between current amplitudes inducted from these two different holding potential (Student’s t-test).

**Fig. 4:**
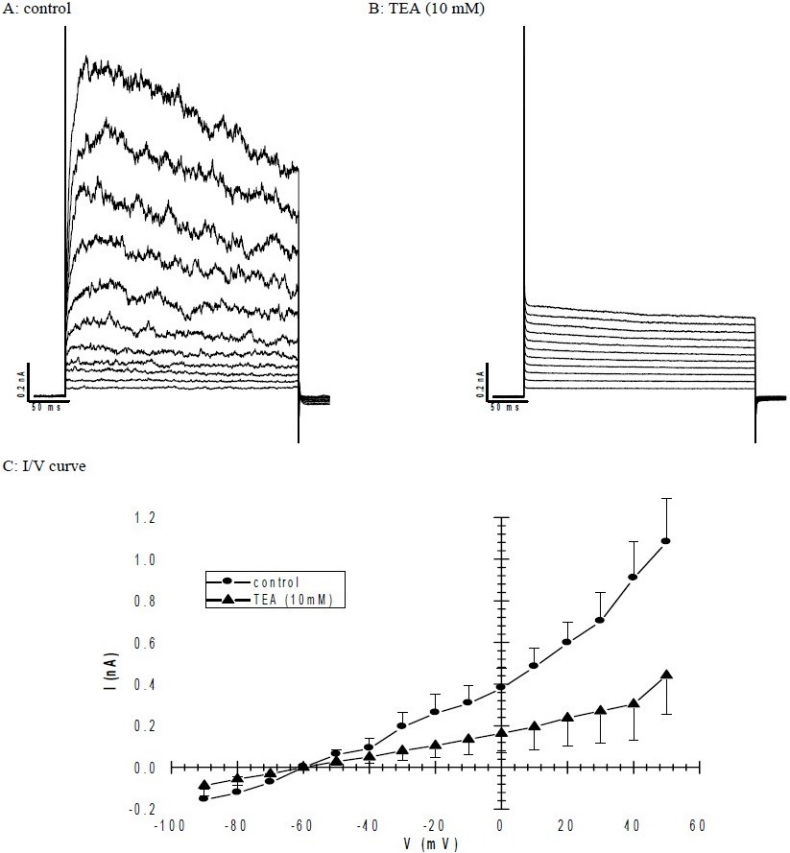
Block of sustained outward current by TEA with 0.2 mM EGTA in KCl recording pipette and Ca^2+^ bath solution. A: Original control current records. B: TEA (10 mM) blocked this current. The sustained current was elicited by depolarising from the holding potential of -60 mV to up to test potential of +50 mV for 300 ms using 10 mV step increments. C: Current-voltage (I/V) curve of the sustained current in control (circle) and presence of 10 mM TEA (tiangle). All the values are mean ± S.E.M (n=4). Mean current amplitudes were measured relative to the holding current at -60 mV.

In this study we have looked at ion channel activity induced by depolarisation from a holding potential of -60 mV which is within the normal range of cardiomyocytes resting membrane potential. A KCl-based pipette solution with low concentration of EGTA (0.2 mM) was used to avoid excessive buffering of the intracellular Ca^2+^ concentration. This is an ideal condition for recording outward K^+^ current. When the holding potential (HP) was - 60 mV a voltage dependent outward current were elicited by square depolarising pulses to positive of -20 mV. This outward current had a turbulence appearance and was sustained for the duration of a 300 ms test-pulse (Fig. 2). The mean amplitude of this current at +30 mV was 0.58 ± 0.15 nA (n=6). More interestingly sometimes the current amplitude was higher at voltages between +10 mV to +30 mV (Fig. 2). The HP had no effect on the activation threshold of the sustained currents. Fig. 3 compares the current-voltage relationship for HPs of -50 and -60 mV. The sustained current was reversibly inhibited from 0.7 ± 0.13 nA to 0.27 ± 0.16 nA by bath applied 10 mM TEA (n=3) (Fig. 4).

This current activation was also dependent on intracellular Ca^2+^. Nevertheless, in some cells TEA did not completely blocked the outward current and some of the outward current still remained.

In contrast, there wasn’t any significant voltage dependent current in the fibroblast cells which were used as feeder cells but in later stage were separated from the stem cells. The voltage induced current amplitude recorded from pure fibroblast cells was very low in comparison with cardiomyocytes. Random recording of K^+^ current from stem cell derived cardiomyocytes revealed that current activation in these cells are not uniform.

**Fig. 5:**
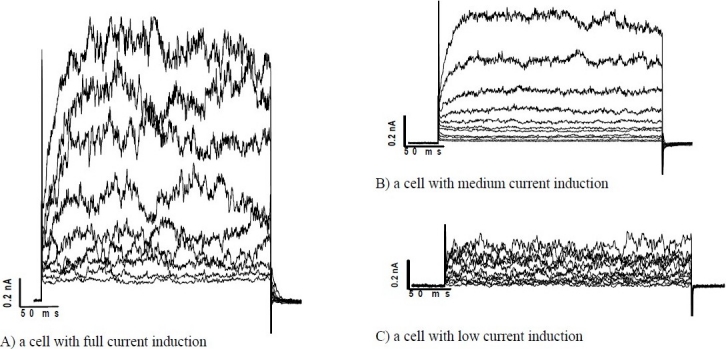
Original sustained outward current recorded with 0.2 mM EGTA in KCl recording pipette and Ca^2+^ bath solution in three different stem cell derived cardiomyocytes. The sustained current was elicited by depolarizing from holding potential of -60 mV to various test potentials ranging from -40 to +50 mV for 300 ms. The fluctuating outward current superimposed on the sustained current is characteristic of the current produced by large conductance Ca^2+^ activated potassium channels. A: The channel activity in this cell is high and produces full current amplitudes (above 1.5 nA when depolarized to +50 mV). B: This cell shows medium channel activity (maximum current amplitues when depolarized to +50 mV is about 0.8 nA). C: Channel activity in this cell is very low and as in this case a tight giga-seal formation needed to see the current, otherwise the leak current would be dominate.

**Fig. 6:**
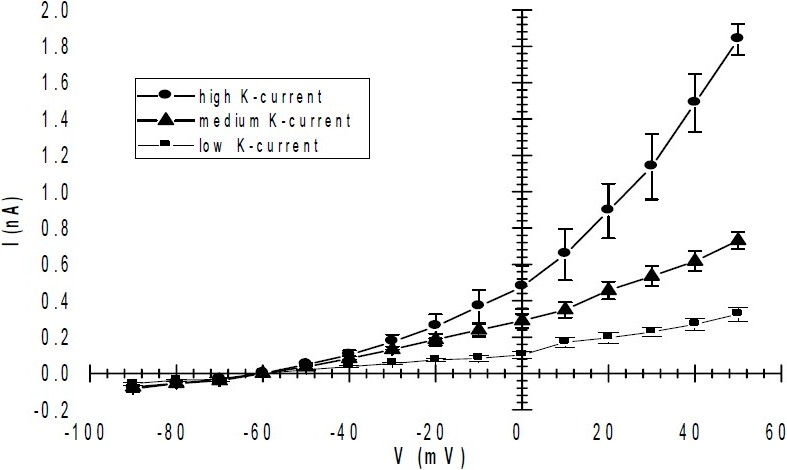
Current-voltage (I/V) curve for outward sustanied current recorded from stem cell derived cardiomyocytes. The sustained current was elicited by depolarizing from holding potential of -60 mV to various test potentials for 300 ms. All the values are mean ± S.E.M (n=7). Mean current amplituedes were measured relative to holding current at -60 mV. According to the maximum induced current amplitued three different class of cells with high, medium and low currents were identified. There are statistically significant differences between current amplitudes in these cells (ANOVA, *P*<0.05). The mean resting membrane potential in these three groups of cells were -44 ± 2.7 mV, -45 ± 3.6 mV and -44 ± 1.7 mV respectively.

When the current amplitude was compared, stem cell derived cardiomyocytes, could be divided in three distinctive groups (Fig. 5). First group elicited high currents rectification and their current amplitude exceed over 1 nA. These cells showed full current activation; however, the number of the cells in the suspensions prepared in the current study were not abundant. Although, the second group of cells did not produced the full current capacities, but still produced a reasonable amount of current for pharmacological evaluation. In the third group which comprised the most abundant cells group, the current amplitude was well below 0.5 nA which only could be recorded with very tight giga-seal. Fig. 6 compares current produced by these three types of cells. In our study, current activation was irrelevant to the cell size or cell resting membrane potential.

## DISCUSSION

Embryonic stem cells are capable of differentiating to a variety of cell tissues including cardiomyocytes (15–18). Differentiation of the stem cells to cardiomyocytes depends on a number of factors including the number of the stem cells to start with for the formation of embryoid bodies and the addition of vitamin C (29–31). This developmental change is accompanied with a great deal of ion channel expression and functions (3). Evaluation of the stem cell derived cardiomyocytes function is mainly accomplished by qualitative assessments such as presence or absence of cell contraction (cell beats) which could be observed in a portion of cell line and could be increased or decreased (32). However, all these findings are subjective comparison rather than quantitative evaluation of the cell function and pharmacological response. Patchclamp technique is an advanced method which can be used to accurately evaluate ion channel function, as well as pharmacologic action of drugs especially in excitable cells including cardiomyocytes.

Although patch pipettes were originally developed for single channel recording, they have advantages over conventional micropipette recording because, firstly, in whole-cell recording there is less damage to the cell and, therefore, it can be used in cells as small as 10 µm in diameter. Secondly, unlike conventional micropipette recording, the pipette-membrane seal is tight (>1 GΩ) and thus there is less leak current. Thirdly, the whole-cell recording pipette has a very low tip resistance (4 Ω) compared with the conventional micropipette tips, which are in excess of 100 MΩ.

If small cells are used, their input resistance is very large compared to the access resistance of the pipette tip so that meaningful electrical measurements can be performed. This allows recording from the whole cell membrane. The whole-cell recording technique allows current-and voltage- clamp of the cell and it was originally used on chromaffin cells (26) but it is now being used for almost every type of cell. The main limitation of whole-cell recording, in terms of voltage control and background noise is the series resistance located in the pipette tip. Therefore, pipettes should be relatively wide and they should have a steep taper (i.e. have low resistance, e.g. 1 MΩ). The dominating source of background noise in the whole-cell recording is likely to be the membrane capacitance in series with the access resistance. The patch pipette for whole-cell recording have tip diameter of 1-2 µm with resistance values of 1-3 MΩ (27,28).

The pipette solution exchanges rapidly with the inside of the cell and so the pipette solution should be similar to the intracellular environment. K^+^ is usually used as the main cation. In addition a HEPES buffer and EGTA-KOH/CaCl_2_ mixture is usually included in order to buffer H^+^ and Ca^2+^, respectively. Some Mg^2+^ ions may also be present. If the buffering capacity is reduced (e.g. <0.1 mM EGTA), the whole-cell recording is more difficult to perform and there is an increased possibility of resealing of the membrane during the course of an experiment.

By using patch-clamp technique an outward K^+^ current was identified in stem cell derived cardiomyocytes. This current was activated by depolarization to -20 mV or more positive. Electrophysiological evidence such as sustained and turbulence current, activation at higher holding potential (-50 mV) indicates the presence of large conductance Ca^2+^ activated K^+^ channel in stem cell derived cardiomyocytes. Ca^2+^ activation also explained why sometimes the outward K^+^ current amplitude is higher in voltages between +10 mV to +30 mV. Because at these voltages Ca^2+^ channels are at their highest activation level, therefore, we have more Ca^2+^ entry and as a result more Ca^2+^ activated K^+^ channels are activated (15,33). In addition, this outwardly rectifying K^+^ current was inhibited by TEA. This is another pharmacological evidence which indicates that K^+^ current recorded here is mainly from large conductance Ca^2+^ activated K^+^ channel. In some cells TEA (10 mM) did not wipe out the outward rectifying current, indicating the presence of different sustained outward current probably a type of delayed rectifier K^+^ current which are also abundant in cardiomyocytes (14,16). In addition, when from holding potential we step back to more negative potential (ie. -70, -80 and -90 mV) a trace of inward current could be seen probably indicating activation of a sort of inward rectifier K channels. Of course these currents are not fully investigated yet but are a subject of research interest in the future.

Fibroblasts are non excitable cells (24), they don’t fire action potential and as expected they didn’t produce any significant current following depolarization. Cardiomyocytes, on the other hand, belong to excitable cells group and not only can fire action potential upon depolarization but also can spread it to the adjacent cells and that is how they produce a unified contraction in the tissue (10–12). Development of ionic current in the stem cell derived cardiomyocytes also indicates that these cells are converted to excitable cells as their ion channels being activated by cell depolarization (3). However, comparison of the current amplitude in various cells indicates that some of the cells have full functional ionic channels. In some other cells although the channel activation is not complete but they still show a great channel activity upon depolarization and they are still on the way of development. Normal cardiomyocytes resting membrane potential is reported to be from about -60 to -80 mV (12,34,35) but our cells had a mean membrane potential of -43 ± 5.4 mV (n=228) which also indicate that some of the cells are not fully developed to mature cardiomyocytes. On the other hand, the pacemaker cells have unstable membrane potential and their maximum diastolic potential is more positive than ventricular cells (12). Cells which had only minor channel activity produced low level of current amplitude indicating that although voltage activated K^+^ channels might be present in these cells but they are not fully developed and they are not functioning, perhaps they are not mature cardiomyocytes yet. Observation of stem cell derived cardiomyocytes contraction under microscope also showed that despite spontaneous contraction in a portion of cell line this contraction does not spread to all the cells and some of the cells showed no sign of contraction. When current amplitude and resting membrane potential of stem cell derived cardiomyocytes were compared, no trend between resting membrane potential and current amplitude produced by the cell was found, therefore, in this study the current amplitude was not related to cell resting membrane potential.

## CONCLUSION

We have shown that Royan B_1_ stem cell derived cardiomyocytes have developed outward K^+^ current which have characteristics of large conductance Ca^2+^ activated K^+^ channels. The channel activity in each cell was precisely quantified by whole cell patch-clamp recording technique. Although it is not possible to measure tension development in cardiomyocytes cell lines but patch-clamp technique is suitable for physiology and pharmacological studies of single cell channel activity and progress of development.
